# A Systematic Review of Treatment of Painful Diabetic Neuropathy by Pain Phenotype versus Treatment Based on Medical Comorbidities

**DOI:** 10.3389/fneur.2017.00285

**Published:** 2017-06-20

**Authors:** Luiz Clemente Rolim, Edina M. Koga da Silva, João Roberto De Sá, Sérgio Atala Dib

**Affiliations:** ^1^Endocrinology Division, Diabetes Center of Universidade Federal de São Paulo (UNIFESP), Escola Paulista de Medicina, São Paulo, Brazil; ^2^Brazilian Cochrane Center of Universidade Federal de São Paulo (UNIFESP), Escola Paulista de Medicina, São Paulo, Brazil

**Keywords:** comorbidity, chronic neuropathic pain, diabetes mellitus, painful diabetic neuropathy, pain phenotype, randomized clinical trial, systematic review

## Abstract

**Background:**

Painful diabetic neuropathy (PDN) is a serious, polymorphic, and prevalent complication of diabetes mellitus. Most PDN treatment guidelines recommend a selection of drugs based on patient comorbidities. Despite the large numbers of medications available, most randomized clinical trials (RCTs) conducted so far have yielded unsatisfactory outcomes. Therefore, treatment may require a personalized approach based on pain phenotype or comorbidities.

**Methods:**

To evaluate whether or not a patient’s pain phenotype or comorbidities can influence the response to a specific PDN treatment, we conducted a systematic review using two different approaches: pain phenotype and associated comorbidities-based treatment.

**Results:**

Out of 45 identified papers, 7 were thoroughly reviewed. We found four RCTs stratified according to pain phenotype with three main results: (1) paroxysmal pain had a better response to pregabalin; (2) the preservation of thermal sensation or nociception anticipated a positive response to the topical treatment of pain; and, (3) after a failure to duloxetine (60 mg/day), the patients with evoked pain or severe deep pain had a better response to association of duloxetine/pregabalin while those with paresthesia/dysesthesia benefited from duloxetine monotherapy (120 mg/day). By contrast, the other three papers provided weak and even contradictory evidence about PDN treatment based on comorbidities.

**Conclusion:**

Although more studies are needed to provide an adequate recommendation for clinical practice, our systematic review has provided some evidence that PDN phenotyping may optimize clinical outcomes and could, in the future, lead to both less empirical medicine and more personalized pain therapeutics.

## Introduction

Diabetic neuropathy (DN) is the most common complication of diabetes mellitus (DM) with a random prevalence ranging from 30 to 50% in individuals with this disease, depending on the method used for diagnosis (1). In addition, DM is the most common cause of polyneuropathy in the western world, as approximately 50% of polyneuropathies are caused by DM (2). Besides that, DN is one of the most impactful chronic diabetes complications, regarding quality of life (QOL), because it is the primary cause of lower limb amputations (85%). It is also directly related to chronic neuropathic pain and its comorbidities, namely, depression, anxiety, and insomnia (1, 2).

Diabetic polyneuropathy is defined as “*a symmetrical, distal and progressive degeneration of the sensorimotor and autonomic peripheral fibers, attributable to metabolic and microvascular changes in consequence of chronic hyperglycemia (DM) and other cardiovascular risk factors*” (1).

Currently, there are about 415 million adults with diabetes in the world (3). Among them, 16–26% suffer from chronic painful diabetic neuropathy (PDN), which means that they present symptoms of neuropathic pain continuous or intermittent for more than 3 months (2, 4). The pain is typically distal, significantly worse at night, and presenting proximal and symmetrical progression: discomfort initially predominant in the toes, feet, and ankles. Regarding the pain phenotype, patients usually describe “burning” associated with a “tingling” sensation, and only approximately 15% present allodynia (4–6).

Thus far, PDN diagnosis and treatment remains problematic and is still open to debate and questioning. Among all PDN patients, unfortunately, up to 39% have never received any treatment for their pain while 12.5% had never even reported their symptoms to doctors (4). Moreover, in most recent therapeutic clinical trials for PDN, only an average of 47% of patients who received duloxetine and pregabalin achieved a 50% reduction in pain scales (5). Another multicentric trial, conducted in France (6) has presented significant data: only 38% of patients correctly diagnosed with PDN were receiving any of the first-line drugs that are consensually recommended.

Finally, in the last 7 years, there has been a proliferation of algorithms and guidelines for the treatment of PDN (7–12) and, in the literature (7), there are at least nine reasons that could explain these facts: (1) high prevalence of DN; (2) social and economic impact of DN; (3) interdisciplinary nature of the disease; (4) the cost of available resources; (5) the lack of reliable information; (6) the lack (and necessity) of data on benefit versus risk; (7) there are at least 34 different drugs recommended to PDN in literature; (8) inconsistent methods to assess the quality of trials; and (9) systematic reviews generally do not include unpublished clinical studies. It is worth noting that there are some contradictory recommendations, such as the ones issued by the American Academy of Neurology (10), the Toronto Consensus (11), and, more recently, the American Diabetes Association position statement about DN (12), which could have been generating confusion among clinicians.

Therefore, there is a considerable global burden caused by PDN and a steady and insoluble demand from both its symptomatic and comorbidities treatment.

## Objectives

Our aim was to assess whether pain phenotype or comorbidities influence the patient’s response to a specific PDN treatment. Two different approaches were utilized to conduct a systematic review, one focusing on treatment based on pain phenotype and the other on comorbidities.

## Methods

We included randomized clinical trials (RCTs) of treatments for PDN in which participants were stratified by pain phenotype or comorbidities in the analysis of therapy efficacy. The literature review was conducted on the Medline database *via* PubMed by using the following index terms: *“clinical trials and painful diabetic neuropathy,” “human diabetic neuropathy and neuropathic pain,” “pain phenotype and randomized study and clinical trials” and “comorbidity and painful diabetic neuropathy.”* We primarily analyzed articles from 2010 to 2016 but included articles from 2009 to 2017 due to high citation index.

We examined titles and abstracts to select the relevant reports. Two authors (LR and ES) independently screened the studies that were identified by the literature search. We retrieved and examined the full text of selected studies for compliance with eligibility criteria. We collected the outcome variables of the intervention effect according to the stratification as the authors presented in the article. Two authors (LR and ES) independently assessed each included study using the Cochrane Collaboration tool for assessing risk of bias (13).

### Definition of Treatment Based on Pain Phenotype

It is a treatment for PDN in which the selection of therapy is stratified by the pain phenotype, the latter meaning pain characteristics regarding signs and symptoms. These features of individual patients or subgroups of patients can increase or decrease the response to a specific treatment (12, 14). Regardless of etiology, neuropathic pain has a strong clinical consistency (14) and can be categorized in five dimensions (pain phenotypes): (1) evoked pain (allodynia or hyperalgesia); (2) paroxysmal pain (electric shock, sharp); (3) deep pain (compression, tightness); (4) superficial pain (burning); and (5) paresthesia and dysesthesia (tingling, brushing).

### Definition of Treatment Based on PDN Comorbidities

It is a treatment for PDN where the selection of therapy is based on concomitant pathologic or disease process. In PDN, there is usually the coexistence of at least one or more disease processes, for instance, cardiovascular disease, anxiety, depression, obesity, autonomic neuropathy, obstructive sleep apnea, peripheral artery disease, nephropathy (dialysis), retinopathy, dementia, and non-alcoholic steatohepatitis.

## Results

Initially, we found 45 articles. We excluded 39 studies because they were neither randomized nor original. However, just one non-randomized study was added because it was a large (2,575 patients) prospective and provided real world-based analysis. Therefore, we identified seven studies that were included in the systematic review: four RCTs based on pain phenotype and three based on comorbidities (two randomized and one observational study).

### Treatment Based on Pain Phenotype

We identified four studies. In the most recent randomized controlled trial (15), the authors found that the combination of moderate doses of imipramine (75 mg/daily) and pregabalin (300 mg/daily) was more effective in the symptomatic treatment of PDN than the monotherapy of one of these drugs, but at the expense of an increased dropout rate, associated with an increased severity and frequency of adverse events (AEs). In this trial, no impact of pain phenotype was registered in the response rates for any of the four groups (imipramine, pregabalin, the combination of both drugs, and the placebo). However, patients with paroxysmal pain tended to respond to pregabalin more often than those without paroxysmal pain (38 versus 10%, respectively, *p* = 0.05).

Another double-blind (without placebo) group (16) examined data from the COMBO-DN trial (17) and studied patients with PDN who lacked a satisfactory response after 8 weeks of monotherapy (duloxetine or pregabalin in doses of 60 and 300 mg, respectively). The authors of this research sought to predict better the analgesic efficacy of different treatment regimens based on painful phenotypes to prescribe a more stratified and personalized treatment for PDN. Patients in an initial duloxetine therapy (60 mg/day) who lacked a satisfactory response particularly benefited from the association of pregabalin (300 mg/day) when presented an evoked or intense deep pain phenotype, while those patients with a paresthesia and dysesthesia phenotype benefited from an increased dose of duloxetine (120 mg/day). On the other hand, patients who received pregabalin (300 mg/day) for initial therapy without a satisfactory response benefited from both duloxetine (60 mg/day) association or increased doses of pregabalin (600 mg/day), regardless of pain phenotype.

A third controlled and randomized trial (18) stratified by pain phenotype was the first to prove that the approach based on pain phenotype could result in a better therapeutic effect. The authors remarked that patients with an evoked pain could be treated with a blocking-sodium-channel agent such as oxcarbazepine. However, only 11% (*n* = 9) of the intention-to-treat population (*n* = 83) of patients had PDN. Nonetheless, in this work, it was the preservation of thermal sensation that anticipated the response to oxcarbazepine, not the evoked pain.

The fourth double-blind, placebo-controlled trial examined the effect of clonidine gel (0.1%) in PDN (19), applied three times per day for 12 weeks on both feet and ankles. Although the group as a whole presented a trend toward improvement on the pain scale (*p* = 0.07), groups that had previously responded to topical capsaicin (0.1%), which means that the patients concerned have a functional nociceptor, significantly improved in pain after the application of clonidine gel. The treatment was safe and did not show any significant AEs.

### Treatment Based on the Comorbidities of PDN

We found three studies. The first was a randomized study (20) that sought to assess the baseline characteristics of demography and comorbidities as predictors of the analgesic effect of duloxetine and pregabalin in patients with PDN. The authors investigated 804 patients from another trial (the multicenter COMBO-DN trial (17)) at baseline and after 8 weeks of monotherapy with duloxetine (60 mg/day) or pregabalin (300 mg/day). No comorbidities or demographic characteristics at the predictor baseline of the analgesic effect were found during the use of the two drugs that were studied. However, both groups of patients reacted better to the medications if they did not have a mood disorder.

The second study that was identified was a 6-month-long prospective, multicenter, observational trial that evaluated 2,575 patients with PDN in the real world (21). In this study, there were no inclusion or exclusion criteria. The purpose of this analysis was to evaluate the impact of comorbidities before initiating pain therapy in the analgesic efficacy and on different pain scales [brief pain inventory (BPI)] (17) of two drug groups: AD (antidepressant: duloxetine) and AC (anticonvulsant: gabapentin, pregabalin). The authors found that 90% of patients had comorbidities: 70% presented hypertension, 37% presented macroangiopathy, 42% presented other chronic pain—especially joint pain or “backache,” and 25% presented depression. Furthermore, patients with depression or with other chronic pain had an analgesic response (BPI) that was greater with duloxetine (60 mg/day) than with AC, although the dosage of gabapentin and pregabalin did not reach the levels that were considered to be effective. The authors concluded that multiple comorbidities are the rule in patients with PDN living in the community and the choice of first-line drugs according to the comorbidity of PDN may be rational.

The third study (22) that we reviewed was a randomized, placebo-controlled trial that examined three first-line drugs (amitriptyline, duloxetine, and pregabalin) for PDN regarding analgesic efficacy, impact on QOL, on polysomnography (PS), and on performance to carry out sensorimotor activities (PSMA). This study presented no statistically significant difference in pain relief during the use of drugs, but there were notable differences in terms of impact on the PS and PSMA. Pregabalin increased sleep time and concomitantly decreased the number of nighttime awakenings and periodic leg movements (PLMs). However, duloxetine and amitriptyline decreased sleep time REM and increased PLMs. On the other hand, pregabalin increased the non-REM phase and only duloxetine significantly increased PSMA.

A summary of findings and their evidence is illustrated in Table 1 with all of the studies that we have reviewed regarding the treatment of PDN stratified by pain phenotype and by comorbidities.

**Table 1:** Summary of findings: the evidence for PDN treatment, based on pain phenotype and comorbidity.

Type of study	*N*	Treatment	Results: phenotype–comorbidity	Conclusion and evidence	Reference
Randomized, multicenter, double-blind, and placebo-controlled	73	4 groups: placebo, pregabalin, imipramine, and combination of both	No impact of pain phenotype on rate response among groups	The percentage of patients with paroxysmal pain tended to respond more often to pregabalin than those without paroxysmal pain (38 versus 10%, respectively, *p* = 0.05)	(15)
Randomized, double-blind, multinational, and stratified by pain phenotype data from COMBO-DN study	339	2 groups (after 8 weeks of duloxetine or pregabalin and without satisfactory response): high dose (duloxetine 120 or pregabalin 600) versus a combination of both (duloxetine 60 + pregabalin 300)	Patients who received duloxetine (60 mg) as initial therapy had: (1) better response to the association of duloxetine + pregabalin with evoked or severe tightness and (2) greater benefit from a high dose of duloxetine (120 mg) with paresthesia–dysesthesia phenotype. In patients with severe pain, there was a tendency to respond better to high-dose monotherapy than association	Patients who received pregabalin (300 mg) as initial therapy benefited from both duloxetine association (60 mg) and a high dose of pregabalin (600 mg), independent of pain phenotype	(16)
Randomized, double-blind, placebo-controlled, and phenotype-stratified study	83	2 groups: placebo and oxcarbazepine	The number of patients needed to treat to obtain one patient with more than 50% of pain relief was 6.9 in the total sample, 3.9 in the evoked pain phenotype, and 13 in the non-irritable nociceptor phenotype. However, it was the preservation of thermal sensation that anticipated oxcarbazepine response	Oxcarbazepine was more efficacious for reliefs of PDN in patients with the irritable versus the non-irritable phenotype. However, in this study, the etiology of neuropathy was heterogeneous: from the total of intention-to–treat (ITT) patient population (*n* = 83), only 11% had PDN (*n* = 9). Therefore, the conclusion should be viewed with caution	(18)
Randomized, double-blind, placebo-controlled, parallel-group, multicentric study	182	2 groups: placebo and topical clonidine	In individuals who felt any level of pain to capsaicin, clonidine was superior to a placebo (*p* < 0.05). In patients with a capsaicin pain rating >2 (0–10), the mean decrease in foot pain was 2.6 for active compared to 1.4 for a placebo (*p* = 0.01)	Topical clonidine gel significantly reduces the level of foot pain in PDN subjects with functional nociceptors. Screening for cutaneous nociceptor function may help to distinguish candidates for topical therapy in PDN	(19)
Randomized, double-blind, multinational, data from COMBO-DN study	804	2 groups (duloxetine and pregabalin) to investigate baseline demographics and comorbidities as predictors of the analgesic effect on PDN	It did not reveal any specific demographic or disease characteristic predictor of analgesia in PDN treatment	Duloxetine and pregabalin were more beneficial for patients without any mood disorder	(20)
Prospective, observational (6 months), multicentric, and real-world study	2,575	2 groups: antidepressive (AD) (duloxetine) and anticonvulsive (AC) (gabapentin, pregabalin)	Better treatment responses with AD versus AC were observed in patients with depression or joint pain. However, the dosage of AC did not reach the effective level	The choice of first-line drugs according to the comorbidity of PDN could be rational. However, it was an observational and non-randomized study	(21)
Randomized, placebo-controlled	83	4 groups: placebo, amitriptyline, duloxetine, and pregabalin	No difference between groups in terms of analgesic efficacy All three treatments were relatively well tolerated, but there was no impact on QOL	Pregabalin increased sleep time and decreased the PLM, whereas duloxetine and amitriptyline decreased sleep time REM and increased PLM. However, it was a short (28-day) dosing study	(22)

*N, number of patients studied; PDN, painful diabetic neuropathy; PLM, periodic leg movement; QOL, quality of life; REM, rapid eye movement*.

As can be seen in Table 2, the studies were generally classified as moderate to high quality. The most biased risks that were verified were the lack of reporting of the randomization and allocation process and the losses in the follow-up period.

**Table 2:** Risk of bias and quality of the RCT included in the systematic review.

Reference	Random sequence generation	Allocation concealment	Blinding participants	Blinding outcome assessment	Incomplete outcome data	Selective reporting	Study quality
(15)	Low risk	Low risk	Low risk	Low risk	Unclear^a^	Low risk	High
(16)	Low risk	Low risk	Low risk	Low risk	High risk	Low risk	Moderate
(17)	Low risk	Low risk	Low risk	Low risk	Unclear^a^	Low risk	High
(18)	Low risk	Low risk	Low risk	Low risk	Unclear^a^	Low risk	High
(19)	Unclear	Unclear	Low risk	Low risk	Low risk	Low risk	Moderate
(20)	Unclear	Unclear	Low risk	Low risk	Low risk	Low risk	Moderate

*^a^Losses in the follow-up, ITT analysis*.

*RCT, randomized clinical trial*.

## Discussion

Currently, consensus guidelines for symptomatic PDN treatment, while still rather empirical, recommend a stepwise approach: drugs from first, second, and third lines (1, 7, 12). Since PDN presents with a high rate of associated comorbidities, as we have shown in this systematic review, several authors recommend that the choice of drugs should be guided by comorbidities. Patients with depression, for instance, could initially receive an antidepressant (AD), while patients with anxiety could preferably receive an anticonvulsant (AC) (1, 7–9). Notwithstanding, such conduct remains based mainly on common sense and physicians’ expertise rather than scientific evidence. As listed in Table 1, for the same drug (duloxetine), there is strong evidence that it can be more beneficial in patients without mood disorder and weak evidence to the contrary; in other words, it could be more efficacious in patients with depression. Nevertheless, in clinical practice, the choice and recommendation of drugs based on comorbidities are understandable and even prudent because the principle of personalized medical treatment is always desirable. Additionally, multiple comorbidities are the rule and not the exception in patients with PDN (21).

Moreover, although a therapeutic approach of neuropathic pain based on pain phenotype has been considered rational, reasonable, and recommended in the last two decades by experts (5, 23), scientific evidence for this proposition in patients with PDN is recent and not yet consensual since guidelines have not yet endorsed this concept. In fact, we have found only four RCTs stratified by the pain phenotype and with three main results: (1) paroxysmal pain had a better response to pregabalin; (2) the preservation of thermal sensation or nociception anticipated a positive response to the topical treatment of pain; and (3) after 8 weeks of duloxetine (60 mg/day) as an initial therapy and a failure to respond to the pain score (BPI) of at least 30%, the patients with evoked pain or severe deep pain phenotype had a better response to association of duloxetine + pregabalin (60 + 300 mg daily, respectively), while those with the paresthesia and dysesthesia phenotype showed greater benefit from a high dose of duloxetine in monotherapy (120 mg/day).

On the other hand, in real-world medicine, two phenomena have caught the attention of different researchers who study neuropathic pain in general and PDN in particular.

First, despite the current advent of new drugs for neuropathic pain and the frequent use of “rational polypharmacy,” a combined treatment, the results of different clinical trials considering pain relief remain rather limited as only 47% of patients in duloxetine and pregabalin trials have achieved a 50% pain reduction (23). Despite there are many possible reasons for these modest results, the lack of an initial approach based on pain phenotype in most clinical trials (22) could be a plausible explanation. Recent evidence has shown that treatment based on pain phenotype is advantageous and seems to be promising for PDN (Table 1). However, in clinical practice, this paradigm shift is more challenging to implement than it initially appears to be, especially for those unskilled or clinical practitioners, since stratification by pain phenotype requires specific training on how to obtain accurate data from the medical history and careful clinical examination (13, 23).

Second, one of the main reasons and criteria for treating patients with neuropathic pain is the intensity of pain. In fact, we know that the worse the pain is, the worse the QOL is; further, the cost of living increases (24). However, this approach has recently been questioned by some researchers (25) who argue that the biggest problem in some trial is that chronic pain is not a simple concept and there is no single and comprehensive approach to assessing the suffering of an individual with pain. They speculate that treatments based on a visual analog scale (0–10) misrepresent the clinical care and have brought about adverse consequences to individuals and general society. These authors propose that multiple measures are needed to assess an individual patient, his/her particular pain, and its causes and consequences (25). Nonetheless, there is some evidence that in patients with PDN and severe pain (16), high-dose monotherapy of pregabalin (600 mg/day) or duloxetine (120 mg/day) seems to be more beneficial than the combination of both in moderate dose (300 + 60 mg/day).

There are some weaknesses in this systematic review. First, the number of included studies was small (seven studies in total) despite the extensive literature search. Second, notwithstanding the fact that the studies which were included presented moderate to high quality (Table 2), they were too heterogeneous to provide an adequate evidence base for clinical practice recommendations. Third, some studies included analyses that were prespecified versus those that were secondary or *post hoc* and were not corrected for multiplicity.

In summary, currently the number and quality of available studies are insufficient to recommend which pain phenotype could have the best response to the individualized PDN treatment. However, evidence is beginning to suggest that the phenotyping of pain may optimize clinical outcomes and could lead to both less empirical medicine and more personalized pain therapeutics. In conclusion, there is a present need to maximize the efficaciousness of the available neuropathic pain medications. Therefore, future randomized studies should have a larger number of patients, stratified by pain phenotype and screened for the nociceptor function in the skin.

## Author Contributions

LCR participated in the conception, searched the literature, extracted and collected data, contributed to data analysis, and wrote and edited the manuscript. EMKS extracted and collected data, and reviewed and edited the manuscript. JRS reviewed and edited the manuscript. SAD participated in the interpretation, review, edition, and writing of the paper. All authors have read and approved the final manuscript.

## Conflict of Interest Statement

The authors declare that the research was conducted in the absence of any commercial or financial relationships that could be construed as a potential conflict of interest. The reviewers, HS and F-YAF, and handling editor declared their shared affiliation, and the handling editor states that the process nevertheless met the standards of a fair and objective review.
